# Multi-frequency ultrasound-assisted cellulase extraction of protein from mulberry leaf: Kinetic, thermodynamic, and structural properties

**DOI:** 10.1016/j.ultsonch.2023.106554

**Published:** 2023-08-07

**Authors:** Li Zhao, Dongyan Ouyang, Xinya Cheng, Xiaotao Zhou, Lebo Lin, Jun Wang, Qiongying Wu, Junqiang Jia

**Affiliations:** a School of Biotechnology, Jiangsu University of Science and Technology, Zhenjiang 212100, China; b School of Grain Science and Technology, Jiangsu University of Science and Technology, Zhenjiang 212100, China

**Keywords:** Ultrasonic extraction, Mulberry leaf protein, Kinetic and thermodynamic parameters, Structural properties

## Abstract

The effects of different extraction methods (traditional extraction, ultrasound extraction, cellulase extraction, and ultrasound-assisted cellulase extraction) on the yield of mulberry leaf protein (MLP) were investigated, and the results revealed that multi-frequency ultrasound-assisted cellulase extraction was the most efficient extraction method. The mechanism of the synergistic extraction method used to efficiently extract protein from mulberry leave was investigated, focusing on the kinetics and thermodynamics of the enzymatic process. The results revealed that kinetic parameters *K_M_* decreased by 14.07% and *k_A_* increased by 5.02%, and the thermodynamic parameters *E_a_*, Δ*H*, and Δ*S* decreased by 44.81%, 48.41%, and 21.12 %, respectively, following the process of multi-frequency ultrasound (MFU) pretreatment. The spectral analysis with fluorescence spectra manifested that ultrasound exposed hydrophobic groups and induced molecular unfolding of MLP. Atomic force microscope showed that ultrasound decreased particle size while increasing flexibility of MLP. The effect of ultrasound increases the binding frequency of cellulase and substrates, resulting in greater affinity between the two and promoting the solubilization of MLP. This study provides a theoretical basis to improve the application prospects of MLP.

## Highlights

• Multi-frequency ultrasound-assisted cellulase can significantly increase the mulberry leaf protein yield.
• Multi-frequency ultrasound promotes cellulase enzymolysis.
• Multi-frequency ultrasound significantly increases the value of the reaction rate constant *k*.
• Multi-frequency ultrasound results in a decrease in the E_a_, Δ*H*, and Δ*S* values.

## 1 Introduction

Mulberry, belonging to the class of *Moraceous* plants, is grown worldwide [1]. Bioactive compounds can be extracted from all parts of a mulberry tree [2]. Different parts of mulberry trees are used to prepare functional foods, such as mulberry leaf noodles, mulberry soup, and mulberry drink powder [3]. Mulberry leaves, in particular, find their use in various fields. They are used not only as food items for silkworms but also as antioxidant, hypolipidemic, and hypotensive agents [4]. Mulberry leaves are rich in proteins (accounting for 17–25% of the dry matter) and amino acids and are considered to be a potential source of vegetable protein [3], [5], [6].

Plant proteins are usually produced following appropriate extraction/precipitation methods, such as alkali extraction/isoelectric precipitation, acid extraction/isoelectric precipitation, and salt extraction/micellar precipitation [7]. Extracting protein from mulberry leaves using traditional methods can be challenging as fibers are tightly bound to the proteins in the leaves [3]. It has been observed that harsh conditions, such as strong acidic and alkaline conditions, affect the nature and structure of the bioactive components in plants [8]. Ultrasound-, enzyme-, microwave-, and pulse-based techniques have been developed in recent years for protein extraction [9]. The combined effects of ultrasound-assisted enzyme extraction and other methods are being investigated worldwide [10]. The findings demonstrate that the recently developed techniques perform better than traditional alkaline extraction methods. In particular, the implementation of ultrasound technology results in the generation of a distinctive cavitation effect that generates high-intensity shear, shockwave and turbulence. This effect significantly improves the biological activity of the compounds, the reaction rate, and the degree of substrate conversion achieved [11]. Wang et al. [12] reported that high extraction efficiency could be achieved when the ultrasound-assisted alkali extraction method was used to extract pea protein. The process was characterized by a short extraction time, and low amounts of water were consumed under these conditions. Jain et al. [13] and Naik et al. [14] reported similar results. Ultrasound frequency significantly affects the rates of chemical reactions [15], and it was observed that the degree of uniformity achieved in energy distribution using the multi-frequency ultrasound (MFU) method was higher than that achieved using the single and dual-frequency ultrasound methods. The cavitation effect generated when the MFU method was used was more effective than that generated when the single and dual-frequency ultrasound methods were used [11]. Ge et al. [16] studied the effect of single, dual, and triple frequencies on the extraction rate of protein from silkworm pupae and reported that the maximum protein extraction rate was recorded when the triple-frequency ultrasound method was used for sample extraction. Similar results were reported by Jin et al. [11]. Enzymes are one of the most effective proteins extractants, and the enzyme-based treatment method is used to improve product yield, modify the texture of the products, and simplify operational steps. These are achieved by degrading cell walls and polysaccharides of plant proteins [17]. Proteases and cellulases, among others, have been used to extract proteins[7]. Naseri et al. [17] used Celluclast®, Shearzyme®, Viscozyme®, and Alcalase® to extract proteins from red seaweed Palmaria palmata. They reported that the maximum extent of protein extraction could be achieved using a combination of Alcalase® and Celluclast® (or Shearzyme®). Shen et al. [18] extracted proteins from tea pulp following alkaline and enzymatic methods and reported that the best extraction results were obtained using a combination of Alcalase and Protamex. The results indicated that the sonication-based and enzymatic techniques were more efficient protein extraction techniques as compared to traditional alkaline extraction methods.

A few studies on the extraction of mulberry leaf protein (MLP) using ultrasound and enzymatic methods have been reported. Sun et al. [1] used the ultrasound cell interference-assisted Osborne method to extract protein from mulberry leaves to study the physicochemical, functional, and antioxidant properties of the protein fractions. Zhu et al. [19] followed the response surface analysis process to optimize the cellulase extraction process. Although the production of MLP by ultrasound has been extensively studied, this protein produced through multi-frequency ultrasound-assisted enzymatic method is rarely reported. In addition, the extraction mechanism of MLP by ultrasound-assisted enzymatic method has not yet been reported. The aim of the present study is, first of all, to study different MLP extraction methods (traditional extraction, ultrasound extraction, cellulase extraction, and ultrasound-assisted cellulase extraction) and to analyze their extraction effects. Additionally, the MLP extraction mechanism of multi-frequency ultrasound-assisted cellulase was investigated by enzymatic kinetics, enzymatic thermodynamics, spectral analysis and atomic force microscope.

## 2 Materials and methods

### 2.1 Materials

Fresh mulberry leaves (protein content: 23.79%; determined following the Kjeldahl method) were harvested in June 2022 from a mulberry garden (West Campus of the Jiangsu University of Science and Technology). Cellulase (50 U/mg) was purchased from Yuan Ye Biotechnology Co. (Shanghai, China).

### 2.2 Extraction of mulberry leaf protein

#### 2.2.1 Cellulase extraction (CE)

The mulberry leaf powder was dissolved in deionized water, the pH of the solution was adjusted to 5.0. The enzyme concentration was maintained at 0.66 g/L, the enzymatic temperature was 40 °C, and the enzymatic treatment time was 30 min. The pH of the solution was adjusted to 9.0 after enzymatic hydrolysis, and the solution was stirred in a water bath for 30 min. Following this, the mixture was centrifuged and the sediment was discarded, and the pH of the supernatant was adjusted to 4.0 until the precipitation of the proteins.

#### 2.2.2 Single-frequency ultrasonic extraction (SUE)

The sample mixture was sonicated using an ultrasonic equipment (Homemade by Jiangsu University of Science and Technology). The ultrasonic parameters were: (ultrasonic frequency: 40 kHz, ultrasonic power: 240 W/L, ultrasonic time: 25 min, solution temperature: 40 °C, material-to-liquid ratio: 1:30 g/mL). After sonication, the proteins were precipitated by Alkaline solubilization and acid precipitation method.

#### 2.2.3 Dual-frequency ultrasonic extraction (DUE)

The extraction method is the same as for SUE, but the ultrasonic frequency is set to 28 kHz + 40 kHz.

#### 2.2.4 Multi-frequency ultrasound extraction (MUE)

The extraction method is the same as for SUE, but the ultrasonic frequency is set to 22 kHz + 28 kHz + 40 kHz.

#### 2.2.5 Multi-frequency ultrasound-assisted cellulase extraction (MUCE)

The extraction process was divided into three steps:

(1) Ultrasonic pretreatment of the sample: ultrasonic frequency: 22 kHz + 28 kHz + 40 kHz, ultrasonic power: 240 W/L, ultrasonic time: 25 min, solution temperature: 40 °C, material-to-liquid ratio: 1:30 g/mL.
(2) Cellulase hydrolysis: After sonication, the pH of the solution was adjusted to 5.0. The enzyme concentration was maintained at 0.66 g/L, the enzymatic temperature was 40 °C, and the enzymatic treatment time was 30 min.
(3) Alkaline solubilization and acid precipitation method for protein extraction.

The control was extracted following traditional alkaline extraction methods. The yields of the MLP were calculated following the method reported by Wang et al. [12].

### 2.3 Kinetics and thermodynamics studies

#### 2.3.1 Effect of the MFU pretreatment on cellulase hydrolysis

The solution of the MFU pretreated mulberry leaf powder was diluted to different concentrations (13, 23, 33, 43, and 53 g/L). Following this, cellulase (0.34, 0.50, 0.66, and 0.82 g/L) was added to the solution, and the mixture was enzymatically digested at different temperatures (20, 30, 40, and 50 °C) over 50 min. Following enzymolysis, the solution was boiled in a water bath at 100 °C for 5 min. The the esulting solution was allowed to cool to 37 °C, centrifuged at 10,000 *g* for 20 min, and the supernatant was used to determine the reducing sugar concentration (dinitrosalicylicic acid method [20]). The saccharification rate was calculated as follows [21]:

(1) $$\mathrm{Saccharificationrate}\%=\frac{\mathrm{CV}}{M}\times 100$$

where C is the concentration of the reducing sugar (mg/mL), V is the volume of the hydrolysate (mL), and M is the sample mass (mg).

#### 2.3.2 Initial reaction rate and kinetic parameters

Reducing sugars are produced during cellulase hydrolysis. The initial rate of cellulase hydrolysis is defined as the amount of reducing sugars released during hydrolysis per unit of time. The rate was calculated as follows:

(2) $$V_{0}=\frac{C_{0}}{5}$$

where V_0_ is the initial reaction rate (g/L*min^−1^), and C_0_ is the concentration of the reducing sugars produced following enzymatic hydrolysis over 5 min (g/L).

The kinetic parameters *K_M_* and *k_A_* were modeled using a previously developed kinetic model [22] as follows:

(3) $$\frac{1}{V_{0}}=\frac{K_{M}}{k_{A}E}\times \frac{1}{S_{0}}+\frac{1}{k_{A}E}$$

where V_0_ is the initial reaction rate (g/L*min^−1^), S_0_ is the substrate concentration (g/L), E is the enzyme concentration (g/L), and *K_M_* and *k_A_* are kinetic parameters.

#### 2.3.3 Kinetics of the enzymatic hydrolysis reaction

A previously developed kinetic model [23] was used to describe the cellulase hydrolysis reaction. A one-solution kinetic model was used for analysis as follows:

（4 $$\frac{dC_{t}}{\mathrm{dt}}=-kt+\ln C_{0}$$

where C_t_ is the concentration of the reducing sugar produced at time t (mg/mL), C_0_ is the initial concentration of the reducing sugar (mg/mL), t is the enzymatic hydrolysis time (min), and k is the reaction rate constant.

（5 $$k=k_{\mathrm{in}}+k_{\mathrm{us}}$$

Here *k_in_* is the rate constant for the reaction induced under conditions of traditional enzymolysis, and *k_us_* is the rate constant of the reaction driven by ultrasound-based conditions.

The reaction rate constant *k* was calculated as follows:

(6) $$\ln Cm-C_{t}=-kt+C_{m}$$

where C_m_ is the maximum concentration of reducing sugar (mg/mL), C_t_ is the concentration of reducing sugar produced at t (mg/mL), and t is the enzymatic hydrolysis time (min).

#### 2.3.4 Thermodynamic parameters

Arrhenius equation and transition state theory [24] were used to obtain the reaction rate constant *k* as a function of temperature. The value was calculated as follows:

(7) $$\ln k=-\frac{E_{a}}{\mathrm{RT}}+\ln A$$

where *k* is the reaction rate constant (1/min), A is the pre-exponential factor (1/min), R is the universal gas constant (8.314 J/mol * K^−1^), T is the Kelvin temperature (K), and *E_a_* is the activation energy (J/mol).

According to Jin et al. [11], the enthalpy of activation (Δ*H*), entropy of activation (Δ*S*), and Gibbs free energy (Δ*G*) can be determined as follows:

(8) $$\Delta H=E_{a}-RT$$

(9) $$\ln\left(\frac{k}{T}\right)=-\frac{\Delta H}{R}\frac{1}{T}+\frac{\Delta S}{R}-\ln\left(\frac{h}{\mathrm{kB}}\right)$$

(10) ΔG=ΔH-TΔS

where k_B_ is the Boltzman constant (1.38 × 10^−23^ J/K), h is the Planck constant (6.6256 × 10^−34^ J * s).

### 2.4 Measurement of structural properties

#### 2.4.1 Scanning electron microscopy (SEM)

The microstructure of the mulberry leaves was examined using a system (Tescan mira3, Czech) operated at an accelerating voltage of 5 kV. Before imaging, samples were mounted onto a specimen holder with the assistance of conductive tape and sputter-coated with gold. The images were recorded at the magnifications of 3000 ×, 5000 ×, and 8000 ×.

#### 2.4.2 Fourier transform infrared spectral *(FTIR)*

The protein samples and a certain amount of KBr were fully ground into a uniform powder in an agate bowl and pressed into flakes. The infrared spectra of MLP were recorded in the wavenumber range 4000–400 cm^−1^ (number of scans: 32 times) using a Fourier transform infrared spectrometer (INVENIO-S, BRUKER OPTICS, Germany) [25]. PeakFit software (SeaSolve Software Inc., Framingham, MA, USA) was used for peak fitting to identify and analyze the secondary structure of MLP [26].

#### 2.4.3 Intrinsic fluorescence spectral (*IFS)*

The MLP samples (2 mg/mL) were dissolved in phosphoric acid buffer (0.01 mol/L) following the process reported by Wu et al. [27]. A fluorescence spectrophotometer (Hitachi F-4600, Japan) was used to record the fluorescence intensities of the protein samples in the range of 300–500 nm. The excitation wavelength and slit width were set at 280 and 5 nm, respectively.

#### 2.4.4 Atomic force microscope (AFM)

20 μL of MLP solution (10 μg/mL) was dropped on the surface of freshly stripped mica substrate and then air-dried overnight at room temperature. An AFM system (Shimadzu spm-9700ht, Japan) was used to examine the surface morphology of MLP.

### 2.5 Statistical analysis

All experiments were carried out in triplicate. All data were analyzed by one-way ANOVA under the significance level of p < 0.05 using SPSS software (SPSS Inc., Chicago, USA).

## 3 Results and discussion

### 3.1 Comparison of extraction methods

**Fig. 1:**
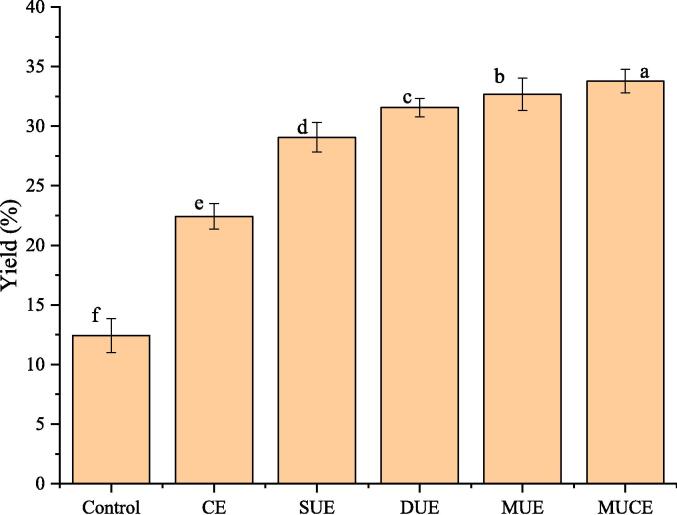
Yield of MLP by different extraction methods. (Control: traditional extraction; CE: cellulase extraction; SUE: single frequency ultrasound extraction；DUE: dual frequency ultrasound extraction；MUE: multi-frequency ultrasound extraction; MUCE: multi-frequency ultrasound-assisted cellulase extraction).

Different methods were used to extract MLP. The results are shown in Fig. 1. It can be observed that good extents of protein extraction were recorded when UE, CE and MUCE methods were used to extract MLP. The same results were shown by Gorguc et al. [7], who reported that the extraction yields obtained using the improved techniques were higher than the extraction yields obtained using traditional alkaline extraction methods. In addition, it was observed that the extraction of MLP by multi-frequency ultrasound had higher yield compared to single and dual frequency ultrasound due to a more uniform energy distribution and greater cavitation [11]. The same results were reported by Ge et al. [16], who found that the combination of ultrasonic frequencies (22/28/40 kHz) was the most effective method in extracting silkworm pupa protein.

It was also observed that MUCE was more efficient than the individual extraction methods. This can be attributed to the fact that ultrasound and enzymes produce a synergistic effect. Ultrasound results in cavitation, turbulence, and shear [14], the compounds degrade into smaller particles, and the enzymes react efficiently with the substrates under these conditions. Cellulase can dissolve the cell walls of plants, promoting the extraction of active ingredients [28]. It can be concluded that MUCE of MLP is an efficient and environmentally friendly extraction method.

### 3.2 Effect of the MFU pretreatment on cellulase hydrolysis

**Fig. 2:**
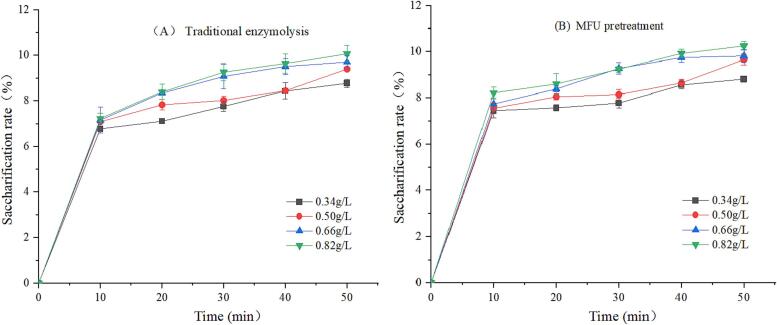
The saccharification rate in traditional enzymolysis (A) and MFU pretreatment (B) at different enzyme concentrations (C_0_ = 33 g/L, pH = 5.0, T = 40 ℃).

**Fig. 3:**
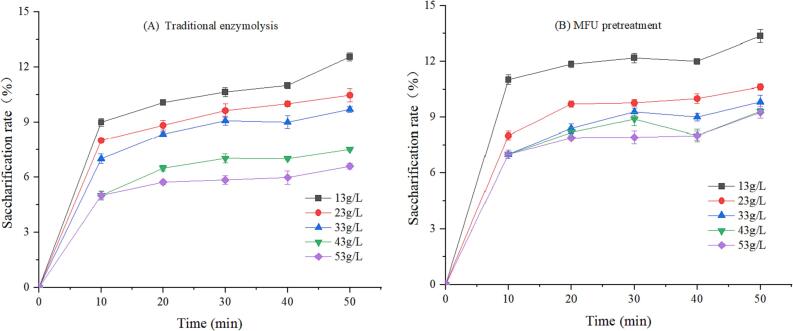
The saccharification rate in traditional enzymolysis (A) and MFU pretreatment (B) at different substrate concentrations (E_0_ = 0.66 g/L, pH = 5.0, T = 40 ℃).

**Fig. 4:**
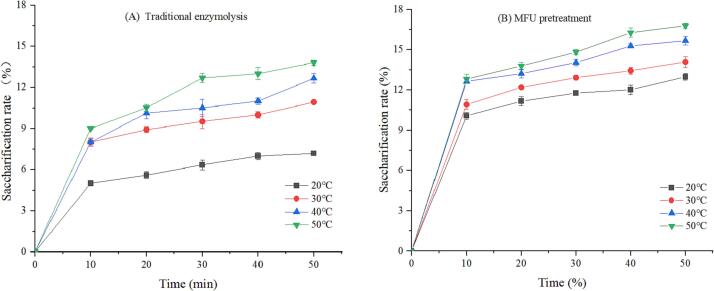
The saccharification rate in traditional enzymolysis (A) and MFU pretreatment (B) at different temperatures (C_0_ = 33 g/L, E_0_ = 0.66 g/L, pH = 5.0).

It was mentioned in the previous paragraph that MUCE of MLP produced higher yields than MUE alone, this experiment explored the effect of MFU pretreatment on cellulase hydrolysis. Cellulase is a complex enzyme that can be obtained from different sources and classified into different types. Different factors influence the properties of this compound, and it is difficult to test the activity of this enzyme. Hence, we analyzed the saccharification rate of the final product (reducing sugar) to express the enzyme activity [21]. Fig. 2, Fig. 3, Fig. 4 shows the saccharification rate of traditional enzymolysis and MFU pretreatment enzymolysis at different enzyme concentrations, substrate concentrations and temperatures for 50 min. It can be observed that the saccharification rate of MFU pretreatment enzymolysis for 50 min was significantly higher than that of traditional enzymolysis under the same conditions. This is due to the fact that the viscosity-reducing effect of ultrasound on the substrate ensures complete decomposition of the mulberry leaves and reduces their intermolecular forces, which promotes the hydrolysis of cellulase and increases the release of MLP [29].

Fig. 2 presents the saccharification rates of traditional enzymolysis and MFU pretreatment enzymolysis at different enzyme concentrations for 50 min. It was observed that the rate of saccharification of powdered mulberry leaf samples increased with an increase in the enzyme concentration. Jin et al. [11] obtained the same trend when they treated corn gluten meal by subjecting the samples to conditions of multi-frequency power ultrasound. This can be primarily attributed to the fact that sonication induces molecular unfolding, which facilitates the reaction between enzymes and substrates[30].

Fig. 3 presents the saccharification rates of traditional enzymolysis and MFU pretreatment enzymolysis at different substrate concentrations for 50 min. The results reveal that the saccharification rate gradually decreases with an increase in the substrate concentration. This indicates that the enzyme–substrate ratio significantly affects the saccharification rate, when cellulase is added in consistent amounts, the rate of saccharification of powdered mulberry leaves negatively correlates with the substrate concentration. This result was consistent with previous studies of ultrasonic pretreatment enzymolysis [11], [31], in which the low substrate concentrations had greater hydrolysate yield than that of the high substrate concentrations. Therefore, the substrate concentration is one of the key factors that affect enzymatic reactions.

Fig. 4 presents the saccharification rates of traditional enzymolysis and MFU pretreatment enzymolysis at different enzymatic temperatures for 50 min. The results reveal that the saccharification rate increases with an increase in the temperature (20–50 °C). The observed phenomenon can be attributed to the high average kinetic energy of the molecules and the high rate of molecular movement at elevated temperatures. We observed that the saccharification rates of the samples pretreated with MFU were higher than those of traditional enzymolysis. This can be explained by the fact that ultrasound pretreatment results in a decrease in the particle size of the substrates, thereby decreasing the strength of the intermolecular forces. Consequently, the average kinetic energy of the moving molecules increases, resulting in an increase in the degree of molecular movement, which eventually results in an increase in the saccharification rate [29]. Therefore, MFU pretreatment can significantly improve the efficiency of cellulase hydrolysis compared to traditional enzymolysis.

### 3.3 Effect of MFU pretreatment on the initial reaction rates and kinetic parameters

The rate of enzymatic reaction can be calculated by analyzing the hydrolysis product content [32]. The concentration of reducing sugars in the cellulase hydrolysate was analyzed to determine the initial rate of hydrolysis. Table 1 presents the initial rates associated with traditional enzymolysis and MFU pretreatment enzymolysis. The data were recorded at different substrate concentrations, and the results revealed that the initial reaction rate increased significantly under conditions of MFU pretreatment. The initial reaction rates recorded under conditions of MFU pretreatment were higher by 20.64%, 6.80%, 8.17%, 17.05%, and 21.41% than the rates recorded under conditions of traditional enzymolysis at the substrate concentrations of 13, 23, 33, 43, and 53 g/L, respectively.

**Table 1:** The initial reaction rate (g/L*min^−1^) of traditional enzymolysis and MFU pretreatment.

Treatments	Substrate concentration(g/L)				
	13	23	33	43	53
Traditional enzymolysis	0.1187 ± 0.001^b^	0.1925 ± 0.012^b^	0.2362 ± 0.078^b^	0.2463 ± 0.006^b^	0.2695 ± 0.035^b^
MFU pretreatment	0.1432 ± 0.003 ^a^	0.2056 ± 0.027 ^a^	0.2555 ± 0.056 ^a^	0.2883 ± 0.049 ^a^	0.3272 ± 0.058 ^a^
Increase (%)	20.64	6.80	8.17	17.05	21.41

Values are the means of three replications ± standard deviation; Different letters above each data in the same column are significantly different (p < 0.05); Values of increase indicated percentage increase of MFU pretreatment to traditional enzymolysis.

**Fig. 5:**
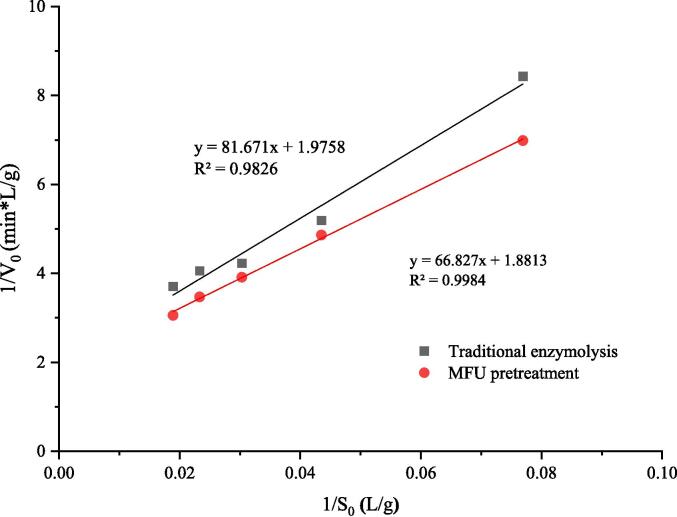
The plots of the reciprocal of the initial reaction rate (1/V_0_) versus the reciprocal of the substrate concentration (1/S_0_) in traditional enzymolysis and MFU pretreatment.

Michaelis constant is a key factor that dictates the kinetic process associated with enzymatic hydrolysis. *K_M_* is the apparent constant, which indicates the affinity of the substrate for the enzyme, and *k_A_* is the apparent decomposition rate, which reflects the frequency of substrate binding to the enzyme [11]. The values of kinetic parameters *K_M_* and *k_A_* can be obtained following the process of linear regression for 1/V_0_ and 1/S_0_ (Table 2). It can be observed that 1/V_0_ and 1/S_0_ exhibit a good linear relationship under traditional enzymolysis and MFU pretreatment enzymolysis. The values of R^2^ were 0.9826 and 0.9984 for the cases of traditional enzymolysis and MFU pretreatment, respectively (Fig. 5). The K_M_ value recorded under conditions of MFU pretreatment was lower by 14.07%, and the *k_A_* value recorded under conditions of MFU pretreatment was higher by 5.02% than the values recorded under conditions of traditional enzymolysis. The decrease in *K_M_* indicates an increase in the affinity between the substrate and the enzyme. This indicates that the affinity of the enzyme increases under conditions of MFU pretreatment. The value of *k_A_* increases under these conditions, indicating that the substrate frequently binds to the enzyme post MFU pretreatment. Qu et al. [33] used ultrasound-assisted preparation methods to analyze the ACE-inhibiting peptides from wheat germ proteins. The results revealed a 6.90% decrease in *K_M_* and a 66.70% increase in the kinetic parameters associated with enzymatic hydrolysis. Li et al. [34] used the ultrasound pretreatment method to analyze beer grain protein and reported a 10.68% decrease in *K_M_* and a 12.11% increase in *k_A_*. The decrease in *K_M_* recorded under these conditions can be potentially attributed to the damage of the substrate structure under the effect of ultrasound. The decrease in *K_M_* values recorded under conditions of enzymatic treatment can be attributed to the fact that sonication disrupts the structure of the substrates resulting in the exposure of a large number of enzyme binding sites. The high pressure, shear, and temperature conditions associated with ultrasonic cavitation promoted the binding of cellulase to the substrate. This eventually resulted in improved reactivity [20]. The results reflect the increase in the *k_A_* values.

**Table 2:** Kinetic parameters k_A_ and K_M_ for traditional enzymolysis and MFU pretreatment.

Treatments	K_M_/(k_A_E) (min)	1/ (k_A_E) (min *L/g)	k_A_ (min^−1^)	K_M_ (g/L)
Traditional enzymolysis	81.671 ± 2.3478 ^a^	1.9758 ± 0.8214 ^a^	0.7668 ± 0.2587^b^	41.3356 ± 3.708 ^a^
MFU pretreatment	66.827 ± 2.6898^b^	1.8813 ± 0.0097^b^	0.8053 ± 0.5465 ^a^	35.5217 ± 4.125^b^
Increase (%)			5.02	−14.07

Values are the means of three replications ± standard deviation; Different letters above each data in the same column are significantly different (p < 0.05); Values of increase indicated percentage increase of MFU pretreatment to traditional enzymolysis.

### 3.4 Effect of MFU pretreatment on the kinetics of the cellulase hydrolysis reaction

**Fig. 6:**
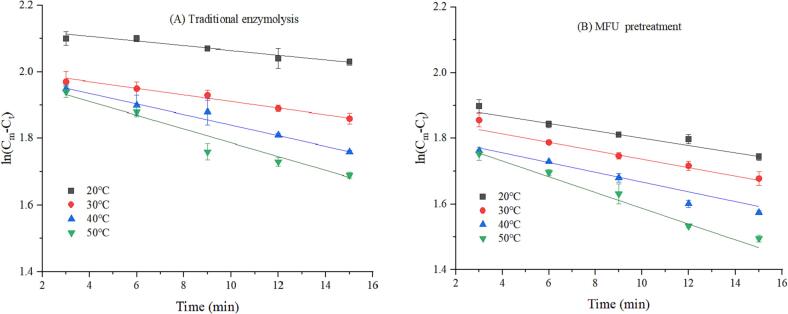
The relationship curves between ln (C_m_-C_t_) and reaction time (t) for traditional enzymolysis (A) and MFU pretreatment (B).

The reaction rate constant *k* is an important variable that dictates the enzymatic hydrolysis process. This parameter is temperature dependent. We investigated the effect of MFU pretreatment on the value of *k* at a substrate concentration of 43 g/L and an enzyme concentration of 0.66 g/L. The pH of the system was maintained at 5.0, and the temperature was varied in the range of 20–50 °C. The curves were generated in the time range of 3–15 min. Fig. 6 presents the ln (C_m_ -C_t_) versus t plots. The plots were generated under conditions of traditional enzymolysis and MFU pretreatment enzymolysis. The rate constant *k* was obtained from the slope of the linear regression plot (Eq. 4). It can be observed that the data fit the linear regression model well (R^2^ >0.93). Therefore, a first-order model can be used to calculate the reaction rate constants. Table 3 presents the reaction rate constants (*k_in_* and *k*) recorded under conditions of traditional enzymolysis and MFU pretreatment in the temperature range of 20–50 °C. It can be observed that the reaction rate constants recorded under conditions of enzymatic hydrolysis increase with an increase in temperature. The same conclusion was obtained by Wu et al.[35], who reported that the *k* value of untreated and ultrasonically pretreated whey proteins increased with increasing temperature. This is because the higher the temperature, the higher the frequency of collisions between the substrate and the enzyme [36]. We also observed that the reaction rate constants recorded under conditions of MFU pretreatment were higher (by 171.01%, 143.43%, 107.01%, and 108.13%) than those recorded under conditions of traditional enzymolysis at all temperatures (in the range of 20–50 °C). Ma et al. [37] investigated the effect of ultrasound on the hydrolysis of alcalase and showed that *k* value was higher than k_in_ value, suggesting that ultrasound improved the catalytic efficiency of alcalase. Ultrasound can generate intense pressure, shear and temperature, changing the structure of the substrate or enzyme, making it easier for the enzyme to bind to the substance and exhibit higher reactivity. Similar results were also reported by Subhedar et al. [20].

**Table 3:** Reaction rate constants and coefficient of determination in traditional enzymolysis and MFU pretreatment at different temperatures.

	Traditional enzymolysis		MFU pretreatment		
T(K)	*k*_in_(min^−1^)	*R^2^*	*k*(min^−1^)	*R^2^*	*k_us_*(min^−1^)
293	0.0069 ± 0.0001^b^	0.9396	0.0187 ± 0.0003 ^a^	0.9621	0.0118 ± 0.0005
303	0.0099 ± 0.0002^b^	0.9877	0.0241 ± 0.0002 ^a^	0.9755	0.0142 ± 0.0003
313	0.0157 ± 0.0006^b^	0.9805	0.0325 ± 0.0001 ^a^	0.9758	0.0168 ± 0.0002
323	0.0209 ± 0.0002^b^	0.9454	0.0435 ± 0.0003 ^a^	0.9855	0.0226 ± 0.0004

Values are the means of three replications ± standard deviation; Different letters above each data in the same row are significantly different (p < 0.05).

### 3.5 Effect of MFU pretreatment on the thermodynamics of cellulase hydrolysis

**Fig. 7:**
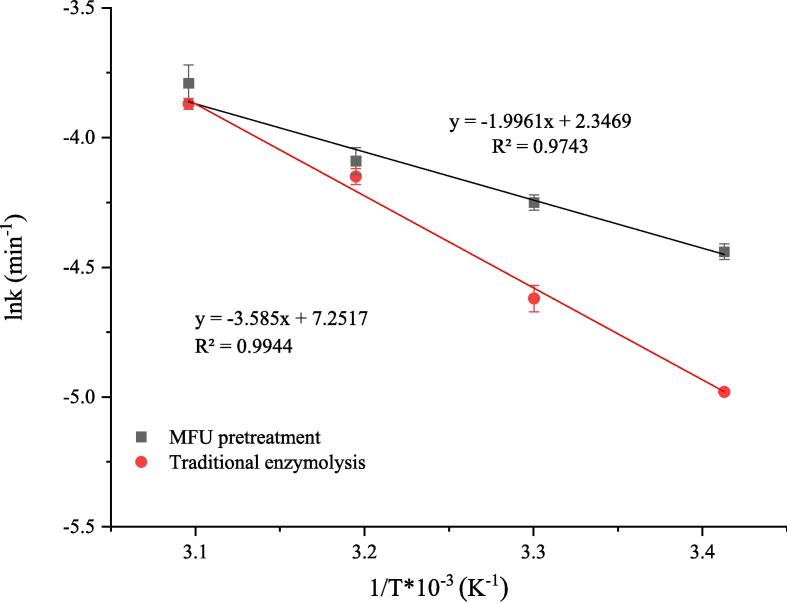
The fitting curves by plotting lnk against 1/T.

**Fig. 8:**
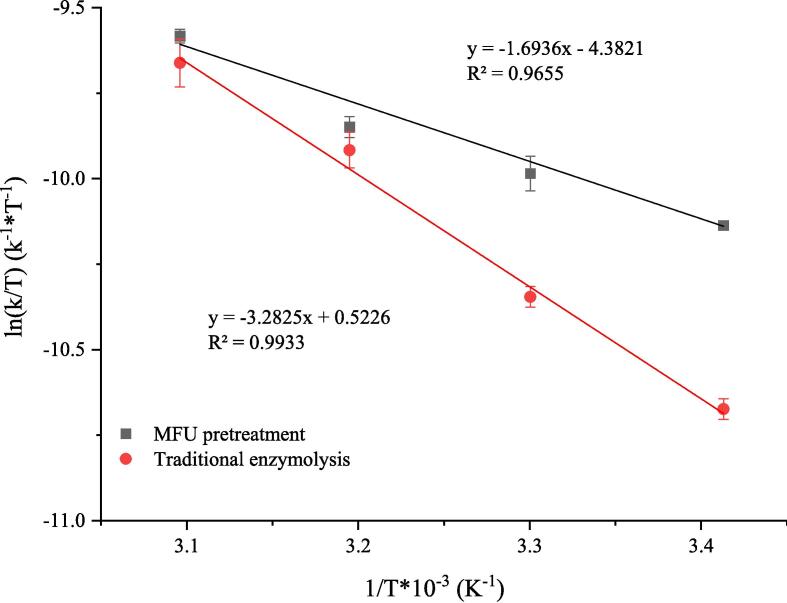
The fitting curves by plotting ln(k/T) against 1/T.

The *E_a_* can be deduced from the slope of the linear plot obtained by plotting lnk against 1/T (Fig. 7). The Δ*H* and Δ*S* of the reaction were calculated using Eyring equation. Fig. 8 displayspresents the ln(k/T) versus 1/T plots. The intercept and slope of the curves were used to determine the Δ*S* and Δ*H* values, respectively. The Δ*G* was computed using Eq. (10). Data on all thermodynamic parameters are presented in Table 4. The lower the *E_a_* value, the faster the reaction. This can be attributed to the fact that the *E_a_* denotes the minimal amount of energy required to move a particle from its ground to its active state. [38]. The *E_a_* recorded under MFU pretreatment enzymolysis conditions decreased by 44.48% (compared to the E_a_ recorded under conditions of traditional enzymolysis). This indicates that a small amount of energy is required for cellulase hydrolysis, and the reaction occurs rapidly under conditions of MFU pretreatment. The results reveal that the substrate binds efficiently to the enzyme under conditions of sonication [39]. The decrease in *E_a_* reveals that the efficiency of the cellulase hydrolysis reaction increases under conditions of MFU pretreatment. This results in a decrease in the amount of energy consumed and an increase in the product yields. Similar results were reported by Golly et al. [40]. It has been previously reported [41] that a positive Δ*H* is indicative of an endothermic enzymatic reaction. Analysis of the data presented in Table 4 reveals that Δ*H* decreased by 48.41% when the samples were subjected to MFU pretreatment conditions. This indicates that the conformation of the substrate changed under the influence of ultrasound, and a large number of particles moved from the ground to the active state under these conditions [32]. A decrease in Δ*S* was observed, indicating a highly ordered arrangement of the substrates and enzymes in the reaction system. Δ*G* is a state function that determines whether a reaction can proceed spontaneously. Δ*G* < 0 indicates that a reaction is spontaneous, and Δ*G* > 0 indicates that a reaction is non-spontaneously. We observed that Δ*G* was > 0 when the reactions proceeded under conditions of traditional enzymolysis and MFU pretreatment enzymolysis in the temperature range of 20–50 °C. This indicates that the nature of the hydrolysis reaction is non-spontaneous. We also observed a non-significant difference in the Δ*G* values recorded under conditions of traditional and MFU pretreatment enzymolysis. This indicated that ultrasound treatment had a negligible impact on the value of Δ*G*. Wali et al. [42] used the sequential dual-frequency ultrasound pretreatment method to extract rapeseed protein and observed that Δ*H*, Δ*S*, and E_a_ decreased by 31.78%, 18.0%, and 29.56 %, respectively. The change in the Δ*G* was insignificant. These results are consistent with our findings. The changes in the thermodynamic parameters associated with enzymatic hydrolysis could be attributed to the increase in the rate of cellulase hydrolysis observed under conditions of ultrasonic pretreatment.

**Table 4:** Thermodynamic parameters in traditional enzymolysis and MFU pretreatment.

				Δ*G*(kJ/mol)			
Treatments	*E*_a_(kJ/mol)	Δ*H*(kJ/mol)	Δ*S*(J/mol·K)	293(K)	303(K)	313(K)	323(K)
Traditional enzymolysis	29.81 ± 1.84^a^	27.29 ± 1.84^a^	−193.11 ± 6.12^a^	83.30^a^	85.23^a^	87.16^a^	89.09^a^
MFU pretreatment	16.55 ± 1.68^b^	14.08 ± 1.68^b^	–233.89 ± 5.45^b^	82.60^a^	84.94^a^	87.28^a^	89.62^a^
Decrease (%)	44.48	48.41	21.12	0.84	0.34	−0.14	−0.59

Values are the means of three replications ± standard deviation; Different letters above each data in the same row are significantly different (p < 0.05); Values of decrease indicated a percentage decrease of MFU pretreatment to traditional enzymolysis.

### 3.6 Analysis of structural properties

#### 3.6.1 SEM analysis

**Fig. 9:**
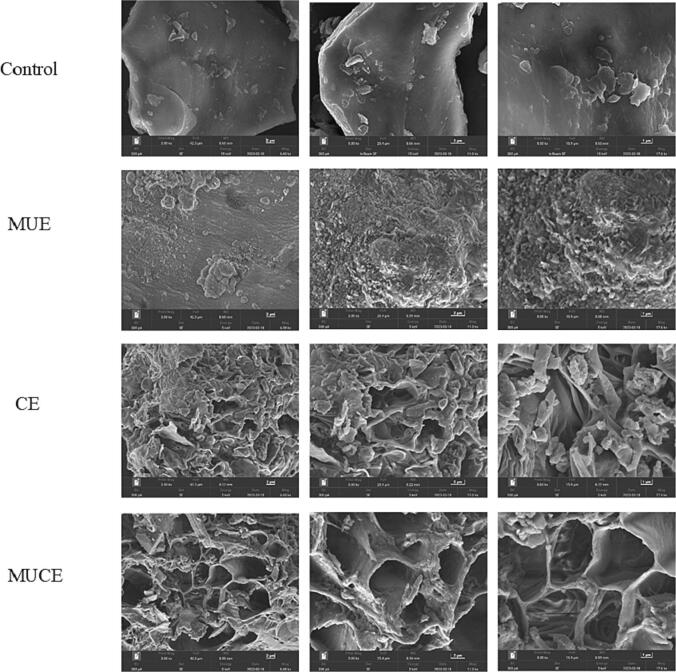
SEM images of the mulberry leaf by different extraction methods. The magnification was 3000×, 5000×, and 8000×, respectively. (Control: traditional extraction; MUE: multi-frequency ultrasound extraction; CE: cellulase extraction; MUCE: multi-frequency ultrasound-assisted cellulase extraction).

The surface morphology of the samples was visualized using the SEM. SEM images of mulberry leaves were recorded at the magnifications of 3000×, 5000×, and 8000× (Fig. 9). Control presents a rigid cellular structure with a smooth surface, and these consist of strongly connected cells. The large mulberry leaf aggregates dissociated into numerous small fragments presenting loosely arranged surfaces when subjected to MFU pretreatment conditions. This finding is consistent with the results of several researchers [12], [43], who found that ultrasound reduces the size of the particles. The maximum deformation effect on mulberry leaves was recorded under conditions of MFU pretreatment. Large perforations were formed on the surface of the leaves subjected to this treatment condition. These leaves presented highly disordered and irregular structures. This can be potentially attributed to the cellular deterioration effect generated when a combination of enzyme and ultrasound treatment methods was used [7].

#### 3.6.2 FTIR analysis

**Fig. 10:**
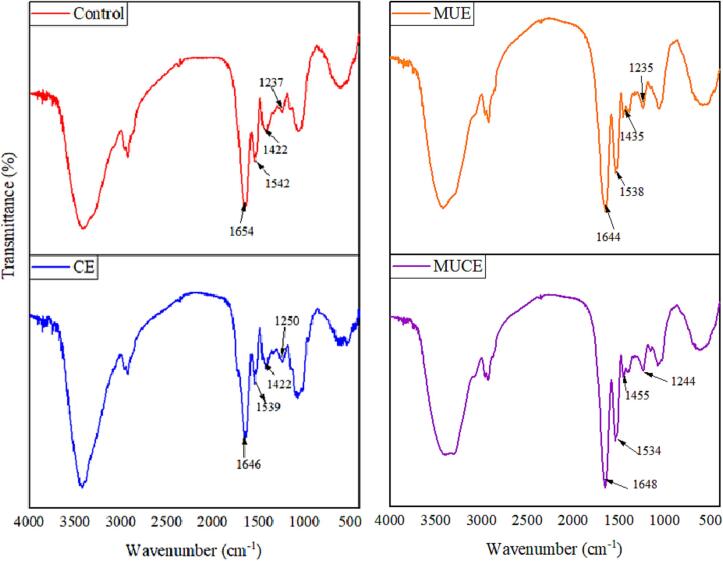
FTIR of MLP by different extraction methods. (Control: traditional extraction; MUE: multi-frequency ultrasound extraction; CE: cellulase extraction; MUCE: multi-frequency ultrasound-assisted cellulase extraction).

FTIR can be used to analyze the secondary structure of MLP. We primarily studied the peak shift in the amide region to arrive at the results. The peak position and shape of the peaks observed in the FTIR recorded for different MLP samples subjected to different treatment conditions were similar (Fig. 10). The amide I peak (1600–1700 cm^−1^) was primarily associated with C = O stretching vibrations, while amide II (1500–1600 cm^−1^) and amide III (1200–1400 cm^−1^) peaks were primarily associated with N-H bending vibrations and C-N stretching vibrations, respectively [25]. The peaks corresponding to the amide region shifted when the samples were subjected to ultrasound and enzyme treatment methods. The amide I peak shifted from 1654 cm^−1^ (control) to 1644 cm^−1^ (MUE), 1646 cm^−1^ (CE), and 1648 cm^−1^ (MUCE), while the amide II peak shifted from 1542 cm^−1^ (Control) to 1538 cm^−1^ (MUE), 1539 cm^−1^ (CE), and 1534 cm^−1^ (MUCE). The amide III peaks shifted from 1237 cm^−1^ (Control) to 1235 cm^−1^ (MUE), 1250 cm^−1^ (CE), and 1244 cm^−1^ (MUCE). These changes can be potentially attributed to the reduction in the number of hydrogen and internal disulfide bonds in the systems. This decrease in the number of bonds can be attributed to the internal breakage of MLP under conditions of sonication and enzymatic hydrolysis [44]. This eventually results in changes in the secondary structure of MLP.

Table 5 presents data on the secondary structure content of MLP; it can be seen that the secondary structure content in MLP changed significantly when the samples were subjected to different treatment conditions. The β-sheet and random coil contents in the samples subjected to conditions of multi-frequency ultrasound extraction were higher than the contents in the control. However, the α-helix content decreased from 25.67% to 17.03%, and the β-turn content decreased from 19.12% to 15.52%. The β-sheet, β-turn, and random coil contents in the samples subjected to conditions of MUCE were significantly higher than the contents recorded for the control samples. However, the α-helix content decreased significantly. This can be potentially attributed to the fact that hydrogen bonds that maintain the structural stability of MLP become weak, and disordered protein structures are formed under the effects of ultrasound and cellulase [44]. The change in the content of the secondary structure also indicates that MUCE alters the structure of MLP. Chandrapala et al. [45] reported an increase in the α-helix content and a decrease in the β-sheet content of whey proteins after ultrasound treatment. Sun et al. [46] also reported that proper ultrasonic pretreatment of soybean isolate proteins could reduce the contents of α-helixes, β-sheets and random coils, while increase β-turns content. These differences can be attributed to the type of protein under study and the treatment environment.

**Table 5:** The secondary structure of MLP.

	Secondary structural contents (%)			
	β-sheets	Random coils	α-Helixes	β-turns
Control	40.44 ± 1.08^d^	14.77 ± 1.36^c^	25.67 ± 1.24^a^	19.12 ± 1.36^c^
MUE	46.49 ± 0.78^a^	20.95 ± 1.25^a^	17.03 ± 1.36^b^	15.52 ± 2.15^d^
CE	41.13 ± 0.63^c^	16.27 ± 0.87^b^	16.16 ± 2.15^c^	26.44 ± 1.63^a^
MUCE	44.93 ± 1.24^b^	16.12 ± 0.69^b^	15.11 ± 1.09^d^	23.77 ± 0.96^b^

Values were mean ± standard deviation. The different superscript letters within the same column mean significant differences (p < 0.05). (Control: traditional extraction; MUE: multi-frequency ultrasound extraction; CE: cellulase extraction; MUCE: multi-frequency ultrasound-assisted cellulase extraction).

#### 3.6.3 IFS analysis

**Fig. 11:**
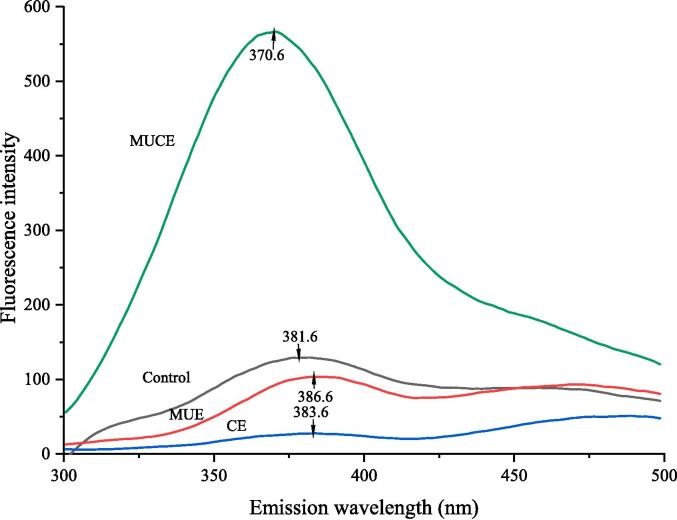
IFS of MLP by different extraction methods. (Control: traditional extraction; MUE: multi-frequency ultrasound extraction; CE: cellulase extraction; MUCE: multi-frequency ultrasound-assisted cellulase extraction).

Tyrosine, tryptophan, and phenylalanine residues in proteins exhibit specific fluorescence properties. The nature of fluorescence depends on the nature of protein folding. The fluorescence spectroscopy technique is often used to analyze the tertiary structures of proteins [47]. As shown in Fig. 11, the fluorescence intensities of the MLP extracted using multi-frequency ultrasound and cellulase showed reduced fluorescence intensity and a red shift in the λ_max_ value compared to the control. The decrease in fluorescence intensity was attributed to the increase in the hydrophobicity of the microenvironment of the tryptophan residue. The increase in hydrophobicity can be attributed to protein fibrillation, and the red shift in λ_max_ can be attributed to the degradation of the proteins into subunits. This results in the exposure of the tryptophan residue to a polar environment [25]. The maximum fluorescence intensity was recorded for the MLP extracted using the MUCE method. This indicated that MUCE could alter the tertiary structure or aggregation states of proteins, disrupting the hydrophobic interactions in the systems. This eventually results in the unfolding of the molecular structure [48].

#### 3.6.4 AFM analysis

**Fig. 12:**
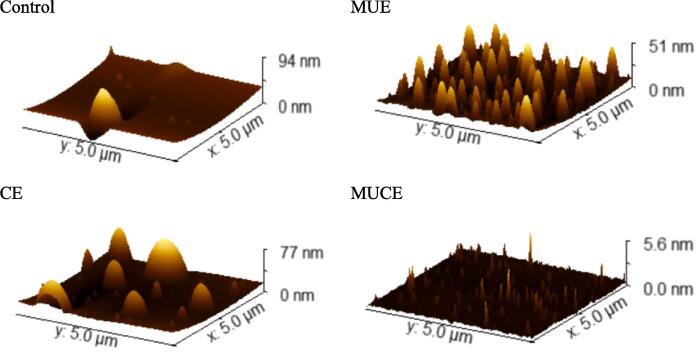
AFM of MLP by different extraction methods. (Control: traditional extraction; MUE: multi-frequency ultrasound extraction; CE: cellulase extraction; MUCE: Multi-frequency ultrasound-assisted cellulase extraction).

AFM was used to determine the microscopic morphology of the protein and understand the structural changes occurring in MLP subjected to different treatment conditions. The results are presented in Fig. 12. The results revealed that the extent of dispersion for the treated samples was higher than the extent of dispersion for the control samples. The particle size decreased significantly when the samples were subjected to different treatment conditions. Similar results were reported by Zhao et al. [49]. This can be potentially attributed to the action of mechanical forces, shear stress, and other effects generated during the ultrasound process. The proteins degrade into smaller particles when subjected to ultrasonic treatment conditions. It was also observed that the roughness of the MLP increased when they were subjected to improved extraction conditions. The maximum degree of increase in roughness was recorded for the samples subjected to conditions of MUCE. The combined effect of ultrasound and cellulase resulted in increased roughness. The protein structure unfolded under these conditions, exposing the hydrophobic groups in the protein to the surface and increasing protein roughness. The results agreed well with the results obtained using the AFM and SEM.

## 4 Conclusion

The results revealed that the yield of MLP increased by 171.76% when MUCE was used for extraction instead of traditional extraction methods. The mechanism associated with MUCE was studied to analyze the kinetics and thermodynamics of the extraction processes. The results revealed that the kinetic parameter *K_M_* decreased by 14.07%, and the kinetic parameter *k_A_* increased by 5.02%, and the thermodynamic parameters Δ*H*, Δ*S*, and *E_a_* decreased by 48.41%, 21.12%, and 44.48%, respectively, compared with traditional enzymolysis, under conditions of MFU pretreatment. SEM analysis revealed that the maximum deformation effect was exerted on mulberry leaves treated following the MUCE. Results obtained using FITR, IFS, and AFM techniques indicated that the combined action of ultrasound and cellulase resulted in a change in the structure of MLP. Thus, it can be inferred that ultrasound and cellulase operate simultaneously and exert a synergistic effect on the process of extraction of MLP. In conclusion, MUCE can be effectively used for the efficient extraction of MLP. The results reported herein provide a theoretical basis to improve the application prospects of MLP to assist in food processing.

## CRediT authorship contribution statement

**Li Zhao:** Conceptualization, Methodology, Software, Investigation, Formal analysis, Writing – original draft. **Dongyan Ouyang:** Data curation, Writing – original draft. **Xinya Cheng:** Visualization, Investigation. **Xiaotao Zhou:** Resources, Supervision. **Lebo Lin:** Resources, Supervision. **Jun Wang:** Software, Validation. **Qiongying Wu:** Supervision. **Junqiang Jia:** Resources, Conceptualization, Validation.

## Declaration of Competing Interest

The authors declare that they have no known competing financial interests or personal relationships that could have appeared to influence the work reported in this paper.
